# p-Cymene inhibits pro-fibrotic and inflammatory mediators to prevent hepatic dysfunction

**DOI:** 10.1515/biol-2022-1054

**Published:** 2025-04-15

**Authors:** Muhammad Atif, Muhammad Nasir Hayat Malik, Tariq G. Alsahli, Muhammad Ali, Waqas Younis, Khalid Saad Alharbi, Sami I. Alzare, Bader Alsuwayt, Tahir Maqbool, Irfan Anjum, Shah Jahan, Abdullah R. Alanzi, Gideon F. B. Solre, Hafiz Muhammad Bilal

**Affiliations:** Faculty of Pharmacy, The University of Lahore, Lahore 54000, Pakistan; Department of Pharmacology, College of Pharmacy, Jouf University, Sakaka, Aljouf 72341, Saudi Arabia; Department of Pharmacology and Toxicology, College of Pharmacy, Qassim University, Buraydah, Al-Qassim, 51452, Saudi Arabia; Department of Pharmacy Practice, College of Pharmacy, University of Hafr Al-Batin, Hafr Al-Batin 31991, Saudi Arabia; Institute of Molecular Biology and Biotechnology (IMBB), The University of Lahore, Lahore 54000, Pakistan; Shifa College of Pharmaceutical Sciences, Shifa Tameer-e-Millat University, Islamabad 44000, Pakistan; Department of Immunology, University of Health Sciences, Lahore 54000, Pakistan; Department of Pharmacognosy, College of Pharmacy, King Saud University, Riyadh, Saudi Arabia; Department of Chemistry, Thomas J. R. Faulkner College of Science and Technology, University of Liberia, Monrovia, Montserrado, Liberia

**Keywords:** p-cymene, HepG2, DEN- CCl4, liver fibrosis, hepatoprotective, TGF-β1

## Abstract

This study evaluated the hepatoprotective potential of p-cymene (p-CYM) against two models of liver damage: ethanol (EtOH)-induced hepatocellular injury and diethylnitrosamine-carbon tetrachloride (DEN-CCl_4_)-induced liver fibrosis (LF). HepG2 cells were treated with p-CYM or silymarin (SIL) before exposure to 10% EtOH in order to induce cellular injury. LF was induced in Sprague–Dawley rats using a single dose of DEN followed by increasing doses of CCl_4_ over 60 days. Rats were treated twice weekly with p-CYM or SIL from day 21 to day 60. Results showed that p-CYM effectively mitigated EtOH-induced cell death in HepG2 cells by enhancing the activity of superoxide dismutase and glutathione reductase. *In vivo* findings revealed that p-CYM attenuated DEN– CCl_4_-induced liver damage by preventing weight loss, improving serum biomarkers (e.g., aspartate transaminase, alanine aminotransferase, alkaline phosphatase, and bilirubin), and reducing liver fibrotic changes. It also decreased the expression of pro-inflammatory and pro-fibrotic markers such as *TNF-α, IL-1β, IL-6, TGF-β1, COL1A1*, and *TIMP-1*. Molecular docking further supported the experimental findings, showing strong interactions between p-CYM and the target proteins. These results indicate that the hepatoprotective effects of p-CYM are likely due to its combined antioxidant, anti-inflammatory, and anti-fibrotic properties.

## Graphical abstract

**Figure:**
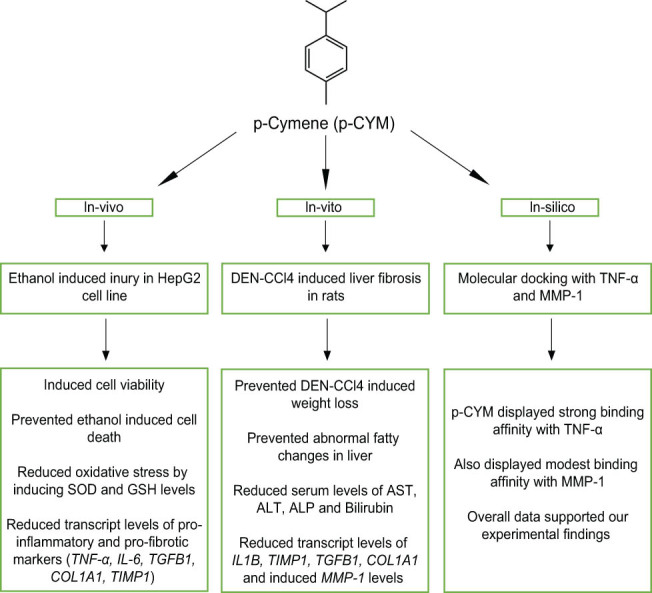

## 1 Introduction

Liver fibrosis (LF) is a condition characterized by the excessive accumulation of extracellular matrix (ECM) proteins, primarily type I and III cross-linked collagens, which form fibrous scars in response to chronic liver injury. This scarring replaces damaged tissue and impairs liver functionality [1]. LF typically results from two primary types of chronic liver damage: hepatotoxic and cholestatic. Cholestatic damage occurs due to bile flow obstruction caused by conditions such as primary and secondary biliary cholangitis, sclerosing cholangitis, and biliary atresia [2]. Hepatotoxic damage, on the other hand, is caused by factors such as alcohol, carbon tetrachloride (CCl_4_), paracetamol, and metabolic syndrome, which lead to steatohepatitis and chronic hepatocyte injury [3,4,5].

Alcohol consumption accounts for approximately 5% of deaths worldwide, with the liver being the primary site for ethanol (EtOH) metabolism [3]. EtOH is metabolized into acetaldehyde, a toxic byproduct, which is further broken down into acetate by acetaldehyde dehydrogenase in liver mitochondria. However, acetaldehyde accumulation and the production of ethyl esters of long-chain fatty acids through non-oxidative metabolic pathways disrupt mitochondrial function, making the liver particularly vulnerable to alcohol-induced damage [5,6,7]. Similarly, CCl_4_ exposure can cause centrilobular hepatic necrosis. Both EtOH and CCl_4_ are metabolized by cytochrome P450 2E1 (CYP2E1), which generates reactive free radicals that contribute to severe hepatotoxicity [4,5,8].

Despite advancements, treatment options for LF remain limited. A variety of drugs, including thalidomide, colchicine, corticosteroids, curcumin, glycyrrhizin, interferons, nitric oxide, resveratrol, silymarin (SIL), and sulfoadenosyl methionine, have gained attention for their anti-fibrotic properties [9,10]. Glycyrrhizin has also shown hepatoprotective effects in patients with sub-acute liver failure, but additional controlled clinical trials are needed [11]. Similarly, thalidomide, resveratrol, and curcumin have demonstrated potential as preventive and therapeutic agents for liver diseases, though their effectiveness across larger populations remains unproven. In cases of end-stage liver disease, liver transplantation remains the only definitive treatment, emphasizing the need for novel therapeutic options to enhance patient outcomes [12].

Medicinal herbs have been used for centuries to treat various diseases, and natural compounds derived from plants continue to attract significant interest in modern medicine [13,14]. One such compound, p-cymene (p-CYM), is an alkyl-substituted aromatic compound with a wide range of pharmacological properties, including antioxidant, anti-inflammatory, antibacterial, antifungal, antiviral, anti-parasitic, anti-diabetic, and anticancer effects [15,16]. A recent study highlighted that p-CYM enhanced the levels of anti-oxidants and reduced inflammatory cytokines in hyperlipidemic rats [17]. Given its reported anti-inflammatory activities and diverse therapeutic potential, p-CYM has been hypothesized to offer hepatoprotective benefits. This hypothesis was tested using a human hepatoma (HepG2) cell line and an LF model to assess its efficacy in mitigating hepatic damage and protecting liver function.

## 2 Materials and methods

### 2.1 Reagents

CCl_4_ (Cat. No. 289116) and SIL (Cat. No. 65666-07-01) were purchased from Sigma-Aldrich Company (St. Louis, MO, USA). Diethylnitrosamine (DEN; Cat. No. 55-18-5) was purchased from Rhawn Chemicals (Shanghai, China) and p-CYM (Cat. No. 99-87-6) from Tokyo Chemical Industry (Tokyo, Japan). All other chemicals used in this research were of standard analytical quality.

### 2.2 Culturing of HepG2 cells

HepG2 cell line was obtained from Cell and Tissue Culture Laboratory (The University of Lahore). Cells were cultured in Dulbecco’s modified Eagle’s medium (DMEM; Cat. No. D5030) supplemented with streptomycin (100 g/ml; Cat. No. S9137), penicillin (100 units/ml; Cat. No. P3032), and 10% fetal bovine serum (Cat. No. F4135) in a humidified incubator at 37°C. Subculturing was done when the cells attained a confluency of 70–80%. Cells were washed with 1× PBS, and adherent cells were detached with 1× trypsin (Cat. No. T4799). The cellular detachment was verified using a phase-contrast inverted microscope. The cell suspension was centrifuged at 2,000 rpm for 5 min, and the obtained cell pellets were resuspended in complete DMEM [18].

### 2.3 Cytotoxicity assessment of p-CYM and EtOH

Cell viability assay was conducted to determine the optimal concentrations of p-CYM and EtOH. Different concentrations of EtOH (1, 3, 5, 8, and 10%) were prepared in complete DMEM, and 1 M stock solution of p-CYM in DMSO (Cat. No. D8418) was prepared. Several dilutions of p-CYM (10, 50, 100, 500, and 1,000 µM) were later formulated from 1 M stock solution. HepG2 cells were seeded on a 96-well plate and incubated at 37℃ overnight. The next day, the medium was removed, and the cells were washed with 1× PBS. Various concentrations of EtOH and p-CYM (100 µl) were introduced into the wells. Cell viability of the treated cells was assessed using the 3-[4,5-dimethylthiazol-2-yl]-2,5 diphenyl tetrazolium bromide (MTT) assay according to the manufacturer’s protocol [19].

### 2.4 Determination of the hepatoprotective effect of p-CYM

HepG2 cells were cultivated on 96- and 6-well plates, and after overnight incubation, they were washed with 1× PBS and pretreated with 100 µl of either SIL (200 µg/ml) or various doses of p-CYM for 24 h. Following p-CYM/SIL treatments, cells were again rinsed with 1× PBS and subsequently treated with 10% EtOH for 24 h. After EtOH injury, cells grown on a 96-well plate were subjected to the MTT, Trypan blue, crystal violet, and PI staining assays. Cells grown on 6-well plates were harvested in TRIZOL reagent for gene expression study, while supernatants were collected for ELISA and anti-oxidant assays [19].

HepG2 cells were divided into the following groups (*n* = 3 in each group):

1. Control: complete DMEM
2. DMSO control: 0.1% DMSO dissolved in complete DMEM
3. Disease control: 10% EtOH dissolved in complete DMEM
4. SIL (200 µg/ml): 200 µg/ml SIL in complete DMEM
5. P-CYM 10 µM: 10 µM p-CYM in complete DMEM
6. P-CYM 50 µM: 50 µM p-CYM in complete DMEM
7. P-CYM 100 µM: 100 µM p-CYM in complete DMEM
8. P-CYM 500 µM: 500 µM p-CYM in complete DMEM

### 2.5 Cell viability assays

In order to calculate the cell viability of pretreated HepG2 cells, MTT and crystal violet assays were performed in which different concentrations of the abovementioned dilutions were tested on cells cultured in 96-well plates.

#### 2.5.1 MTT assay

Pretreated cells were washed with 1× PBS followed by 3–4 h of incubation with 100 µl of DMEM and 25 µl of MTT (Cat. No. M5655) solution. Formazan crystals were solubilized with 10% sodium dodecyl sulfate (SDS), and absorbance at 570 nm was measured using a microplate reader. Percentage cell viability was calculated from the mean absorbance values [18].

#### 2.5.2 Crystal violet assay

Pretreated cells were rinsed with 1× PBS and treated with a mixture of 0.1% crystal violet dye and 2% EtOH, followed by incubation for 15 min at room temperature (RT). Wells were thoroughly washed with 1× PBS, and the dye was carefully disposed of to prevent cells from lifting out of the wells. The stain was then solubilized by adding 100 µl of 1% SDS to each well. Finally, the absorbance was measured at 595 nm using a microplate reader [18].

### 2.6 Dead cell detection

For dead cell detection, a trypan blue assay was performed.

#### 2.6.1 Trypan blue staining

Trypan blue reagent was used to distinguish between live and dead cells. Briefly, pretreated cells were washed three times with 1× PBS and subsequently stained with trypan blue (Cat. No. T6146). The blue-stained cells were designated as dead, which were counted using a compound microscope.

### 2.7 Antioxidant assays

#### 2.7.1 Glutathione reductase (GSH) assay

GSH levels in the samples were quantified using the Bioassay Technology Laboratory ELISA Kit (Cat. No. EA0142Hu). Reagents, standard solutions, and samples were prepared according to the kit’s instructions and equilibrated to RT prior to use. For the assay, 50 µl of the standard solution was added to the standard wells, and 40 µl of the sample was added to the sample wells. Subsequently, 10 µl of anti-GSH antibody was added to each well, followed by 50 µl of streptavidin-HRP to the sample wells. The contents were mixed thoroughly, sealed with a plate sealer, and incubated at 37°C for 60 min. After incubation, the sealer was removed, and the plate was washed five times using 300 µl of wash buffer per well, with each wash lasting 30 s to 1 min. Following the washes, 50 µl of substrate solution A and 50 µl of substrate solution B were sequentially added to each well. The plate was then incubated in the dark for 10 min at 37°C. After incubation, 50 µl of stop solution was added to each well, resulting in an immediate color change from blue to yellow. The optical density (OD) of each well was measured immediately using a Bio-Rad microplate ELISA reader (Model PR4100) set to a wavelength of 450 nm [20].

#### 2.7.2 Superoxide dismutase (SOD) assay

SOD was measured using the ELISA Kit from Bioassay Technology Laboratory (Cat. No. E4502Hu), and the same procedure was adopted, as described for the GSH assay.

### 2.8 Animals used

Male Sprague–Dawley rats weighing 150–200 g were purchased from the University of Veterinary and Animal Sciences (Lahore, Pakistan). Animals were kept under standard conditions (temperature: 22 ± 2°C and humidity: 60 ± 10%) with a 12 h light and dark cycle at the Animal House of the Faculty of Pharmacy (The University of Lahore). All the animals were given free access to food and water throughout the adaptation and experimental period. This study was performed in accordance with ARRIVE guidelines.


**Ethical approval:** The research related to animal use has complied with all the relevant national regulations and institutional policies for the care and use of animals and has been approved by the Institutional Research Ethics Committee (IREC) of the Department of Pharmacology, Faculty of Pharmacy, The University of Lahore, Lahore, Pakistan (Approval Number: IREC-2022-46).

### 2.9 LF experimental design

Animals were randomly divided into five groups (*n* = 4 in each group): control, disease (CCl_4_), standard (SIL), and treatment (p-CYM) groups. The control group received twice a week intraperitoneal (i.p.) injection of olive oil (0.5 ml/kg). LF was induced using a single dose of DEN, followed by increasing doses of CCl_4_ for 60 days (D). Briefly, a day after DEN administration, CCl_4_ (0.5 ml/kg) was injected intraperitoneally (i.p.) twice a week for 40D. On D41, animals were treated twice a week with 1 ml/kg of CCl_4_ for 18 D, i.e., D41–D58, which was later increased to 2 ml/kg for the last 2 days, i.e., D59–D60. To investigate the protective effects of p-CYM and SIL, animals were treated with SIL (100 mg/kg) and p-CYM (50 and 100 mg/kg) twice a week from D21 to D60. On D60, the body weights of animals were measured, and later, they were sacrificed by intraperitoneal injection of pentobarbital sodium (200 mg/kg) to collect blood and liver samples for subsequent biochemical, histopathological, and RT-qPCR analyses. Care was taken to minimize the suffering of animals.

### 2.10 Biochemical and histopathological analyses

Serum aspartate transferase (AST), alanine aminotransferase (ALT), alkaline phosphatase (ALP), and bilirubin levels were measured using standard ELISA kits. Liver samples fixed in 10% buffered formalin were sectioned using the paraffin embedding technique and stained with hematoxylin and eosin for histopathological analysis.

### 2.11 Real-time PCR analysis

Total RNA was isolated from liver samples using the Trizol method, which was later reverse-transcribed using the WizScript cDNA Synthesis Kit (Wizbio solutions, New Mexico, USA; Cat. No. W2202). The relative transcript levels of genes were measured by the ΔΔ*C*_T_ method using Zokeyo 2xSYBR Green qPCR mixture (Cat. No. HPR012-01). The following PCR conditions were used to measure the CT values: initial denaturation was carried out at 94°C for 2 min, followed by 40 cycles of denaturation at 94°C for 1 min, annealing at 60°C for 30 s, and elongation at 72°C for 15 s. Hypoxanthine guanine phosphoribosyltransferase (HPRT) was used as an internal standard. A list of primers (Macrogen, South Korea) used in the study is provided in Table S1.

### 2.12 Molecular docking analysis

#### 2.12.1 Retrieval of tumor necrosis factor-alpha (TNF-α) and matrix metalloproteinase-1 (MMP-1) structures from Protein Data Bank

The three-dimensional (3D) structures of human TNF-α and MMP-1 were obtained from the Protein Data Bank (PDB) using PDB IDs 2AZ5 and 4AUO, respectively. The target proteins were prepared for docking analysis using the Autodock Tool program. Proteins were reduced in energy, given Gasteiger charges, and saved in a pdbqt format. Discovery Studio 4.1 Client (2012) was used to generate Ramachandran plots. VADAR 1.8 was used to access the protein architecture and statistical percentages of helices, β-sheets, coils, and turns [21].

#### 2.12.2 Ligand molecular docking

P-CYM was drawn in Discovery Studio Client and saved in a pdb format as a ligand. The most stable conformation of the ligand was prepared using Autodock Tools. The Kolman and Gasteiger charges were added before the ligand was saved in a pdbqt format. The synthetic ligand (p-CYM) was subjected to a molecular docking experiment using PyRx’s virtual screening tool and the Auto Dock VINA Wizard method [22].

### 2.13 Statistical analysis

Data of 3–4 biological replicates were presented as the mean ± SD and were analyzed by one-way ANOVA followed by Tukey’s multiple comparison test. Statistical analyses were performed using Graph Pad Prism 8.0 software (Graphpad Software, Inc., San Diego, USA). A probability of less than 0.05 was considered significant. The level of significance was expressed as *** ≤ 0.001, ** ≤ 0.01, * ≤ 0.05.

## 3 Results

### 3.1 P-CYM treatment did not alter the viability of HepG2 cells

The cytotoxicity of p-CYM in HepG2 cells was assessed using the MTT assay to determine its safe and tolerable concentrations. HepG2 cells were treated with increasing concentrations of p-CYM (10, 50, 100, 500, and 1,000 µM) for 24 h to evaluate its impact on cell viability. The assay results demonstrated that p-CYM did not significantly affect cell viability at lower concentrations, specifically 10, 50, 100, and 500 µM, indicating that these doses are non-toxic to the cells and can be considered safe for further experimental use. However, at a concentration of 1,000 µM, p-CYM exhibited cytotoxic effects, as evidenced by a significant reduction in cell viability. These findings suggest that while p-CYM is generally well tolerated at moderate doses, high concentrations may compromise cellular health, emphasizing the importance of dose optimization for its potential therapeutic applications (Figure S1).

### 3.2 Cytotoxic effects of EtOH in HepG2 cells

To determine the toxic effects of EtOH on HepG2 cells, the cells were exposed to increasing concentrations of EtOH (ranging from 1 to 10%) for a duration of 24 h. Cell viability was then assessed to evaluate the extent of toxicity. At lower concentrations, specifically between 1 and 4%, EtOH did not induce any significant reduction in cell viability. This suggests that these concentrations are relatively safe and do not cause notable cellular damage. However, as the concentration of EtOH increased, a gradual decrease in cell viability was observed. At 6% EtOH, the reduction in cell viability was minimal, but it became more pronounced at 8%. The most substantial decline in cell viability occurred at the highest concentration tested, 10% EtOH, where cell viability dropped by over 50%. This indicates that EtOH has a dose-dependent toxic effect on HepG2 cells, with significant cellular damage occurring at concentrations of 8% and 10%. These findings suggest that high concentrations of EtOH can induce severe cytotoxicity, potentially through mechanisms like oxidative stress or disruption of cellular functions (Figure S2).

### 3.3 P-CYM protected against EtOH-induced toxicity in HepG2 cells

**Figure 1:**
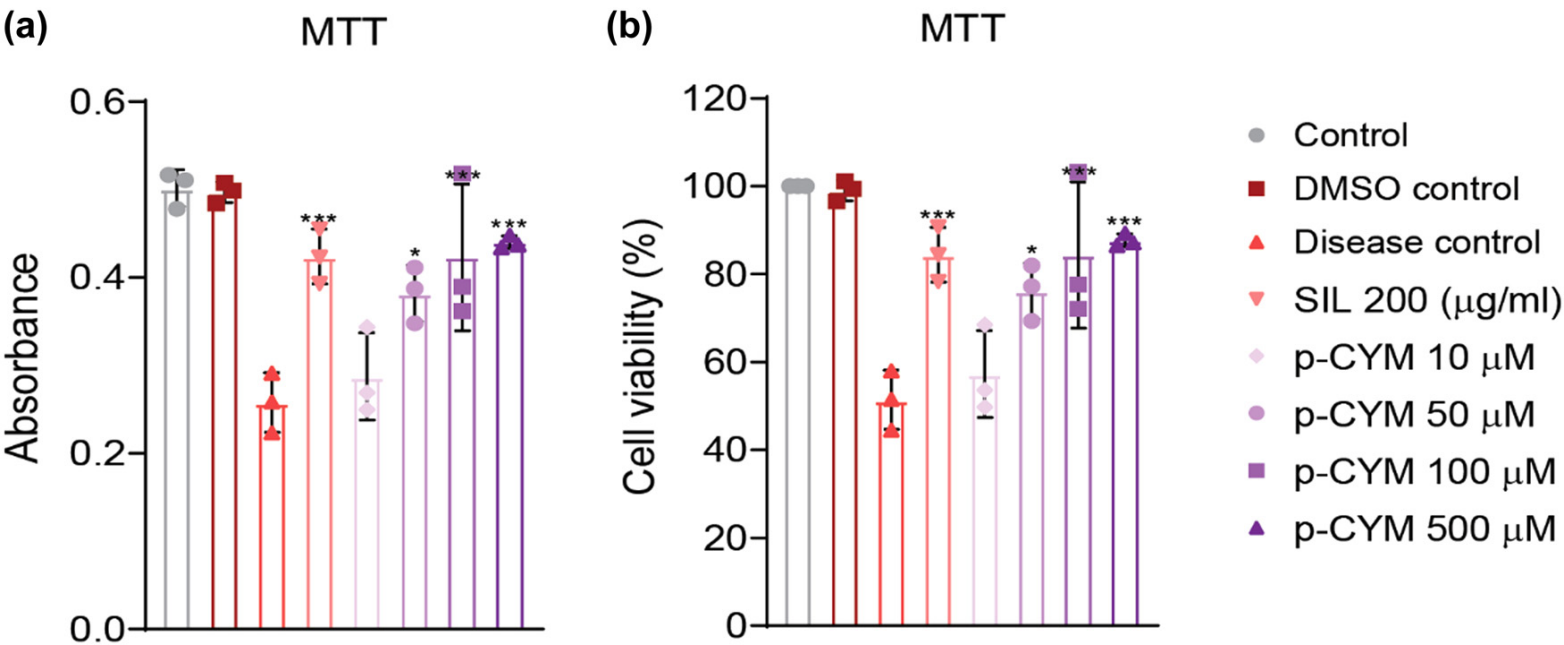
Cytoprotective effects of p-CYM against EtOH-induced damage in HepG2 cells. (a) The absorbance of MTT dye was measured. (b) Percentage cell viability was measured from the absorbance values. Pre-treatment of p-CYM (50, 100, and 500 µM) significantly reduced EtOH toxicity compared to the disease control group. *** ≤ 0.001, ** ≤ 0.01, * ≤ 0.05 (treated groups vs disease group); one-way ANOVA followed by Tukey’s multiple comparison test; *n* = 3.

The cytoprotective effects of p-CYM against EtOH-induced cell injury were investigated by treating HepG2 cells with various concentrations of p-CYM (10, 50, 100, and 500 µM) prior to EtOH exposure. EtOH exposure significantly reduced cell viability by approximately 50% compared to the p-CYM- and SIL-treated groups, demonstrating the toxic impact of EtOH on liver cells. However, pretreatment with p-CYM at concentrations ranging from 10 to 500 µM resulted in a dose-dependent attenuation of EtOH-induced cytotoxicity. The protective effect of p-CYM was most prominent at 500 µM, where it demonstrated cytoprotective effects comparable to those of SIL, a well-known hepatoprotective agent (Figure 1).

To further assess the impact of p-CYM on cell viability, a crystal violet assay was performed, showing a reduction in cell viability of around 40% upon exposure to EtOH, which aligns with the initial findings. However, p-CYM treatment was able to effectively restore cell viability in a significant manner, suggesting its potential to counteract EtOH-induced damage. Interestingly, the cellular growth observed in the p-CYM-treated group was even more pronounced than in the SIL-treated group, indicating that p-CYM might have a stronger or more favorable effect on promoting cell recovery and proliferation after ethanol-induced injury. These results highlight the strong protective and restorative capabilities of p-CYM, positioning it as a promising agent for preventing or mitigating liver damage caused by EtOH (Figure S3).

### 3.4 P-CYM prevented EtOH-induced cell death

**Figure 2:**
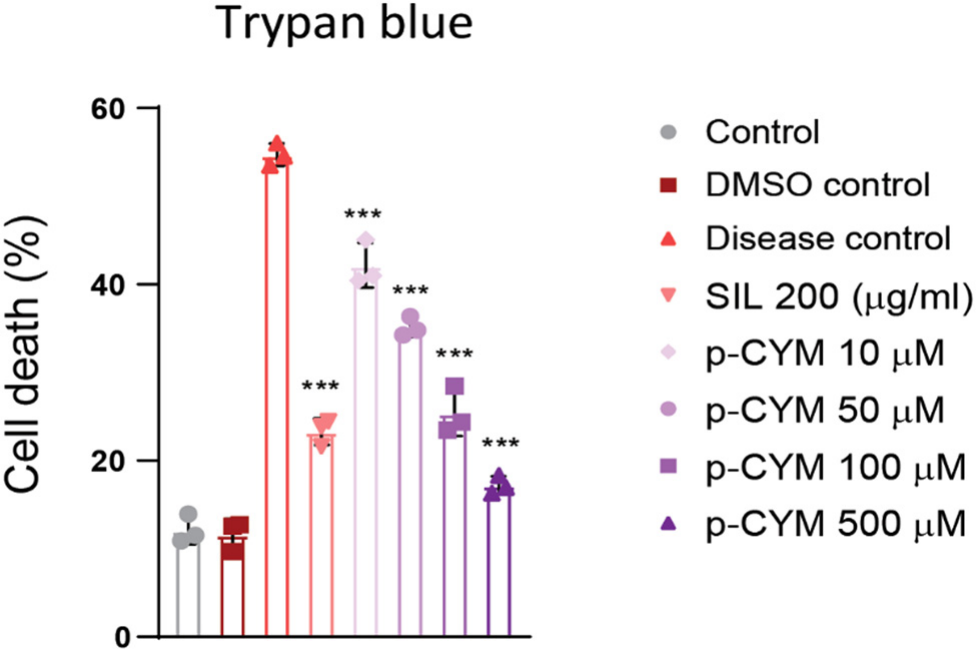
P-CYM reduced EtOH-induced cell death. Trypan blue staining showed an increased cell death with EtOH treatment. P-CYM and SIL treatments significantly reduced EtOH-induced cell death in HepG2 cells. *** ≤ 0.001 (treated groups vs disease group); one-way ANOVA followed by Tukey’s multiple comparison test; *n* = 3.

The Trypan blue assay was used to assess cell death in HepG2 cells following exposure to EtOH. The results showed that EtOH induced cell death in more than 50% of the HepG2 cells, confirming its toxic effects on the cells. However, pretreatment with p-CYM demonstrated a dose-dependent reduction in cell death compared to the disease control group. At concentrations of 500 µM, its cytoprotective effects were even more pronounced than those observed in the SIL-treated group. This indicates that p-CYM not only reduced cell death but also showed superior efficacy in protecting HepG2 cells from EtOH-induced toxicity, highlighting its potential as a more effective therapeutic agent compared to SIL (Figure 2).

### 3.5 P-CYM attenuated EtOH-induced oxidative stress and transcript levels of inflammatory and fibrotic modulators

**Figure 3:**
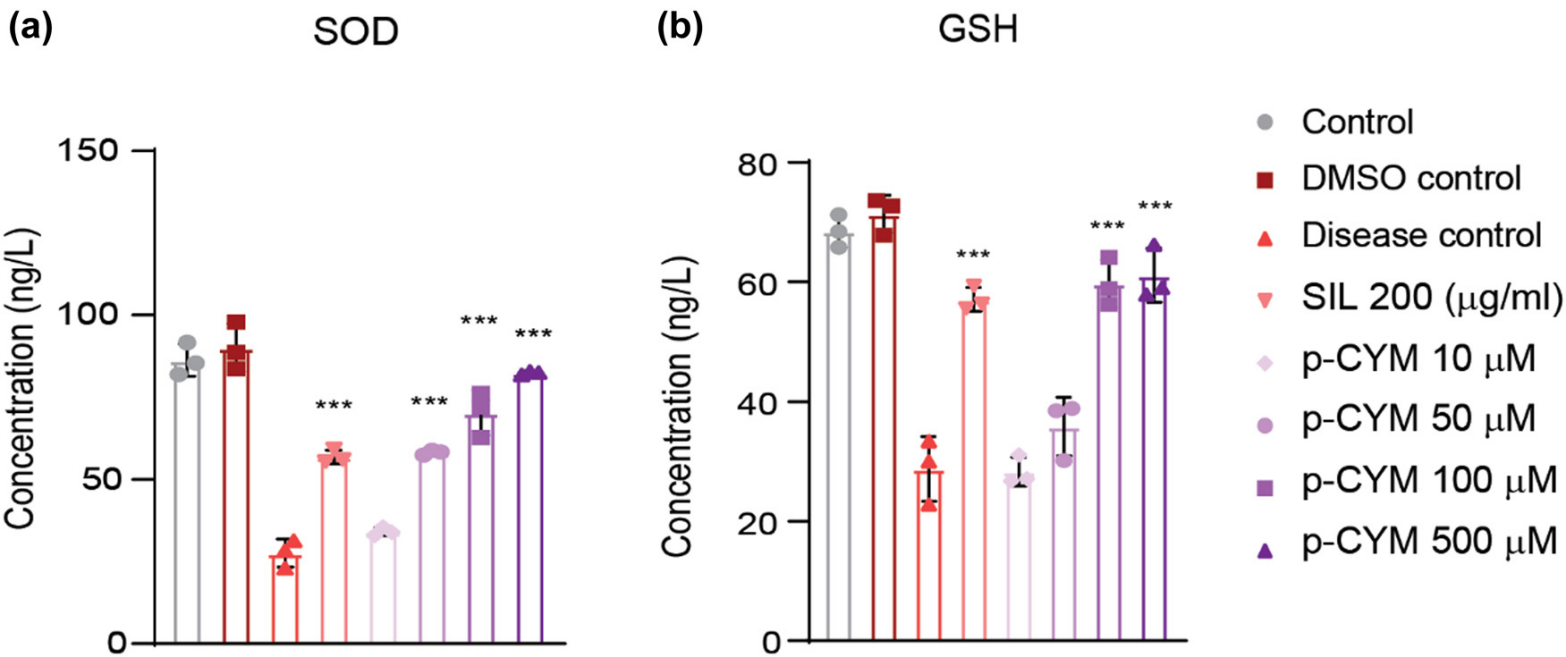
P-CYM prevented EtOH-induced oxidative stress by inducing anti-oxidants. P-CYM and SIL induced the levels of SOD (a) and GSH (b). *** ≤ 0.001 (treated groups vs disease group); one-way ANOVA followed by Tukey’s multiple comparison test; *n* = 3.

**Figure 4:**
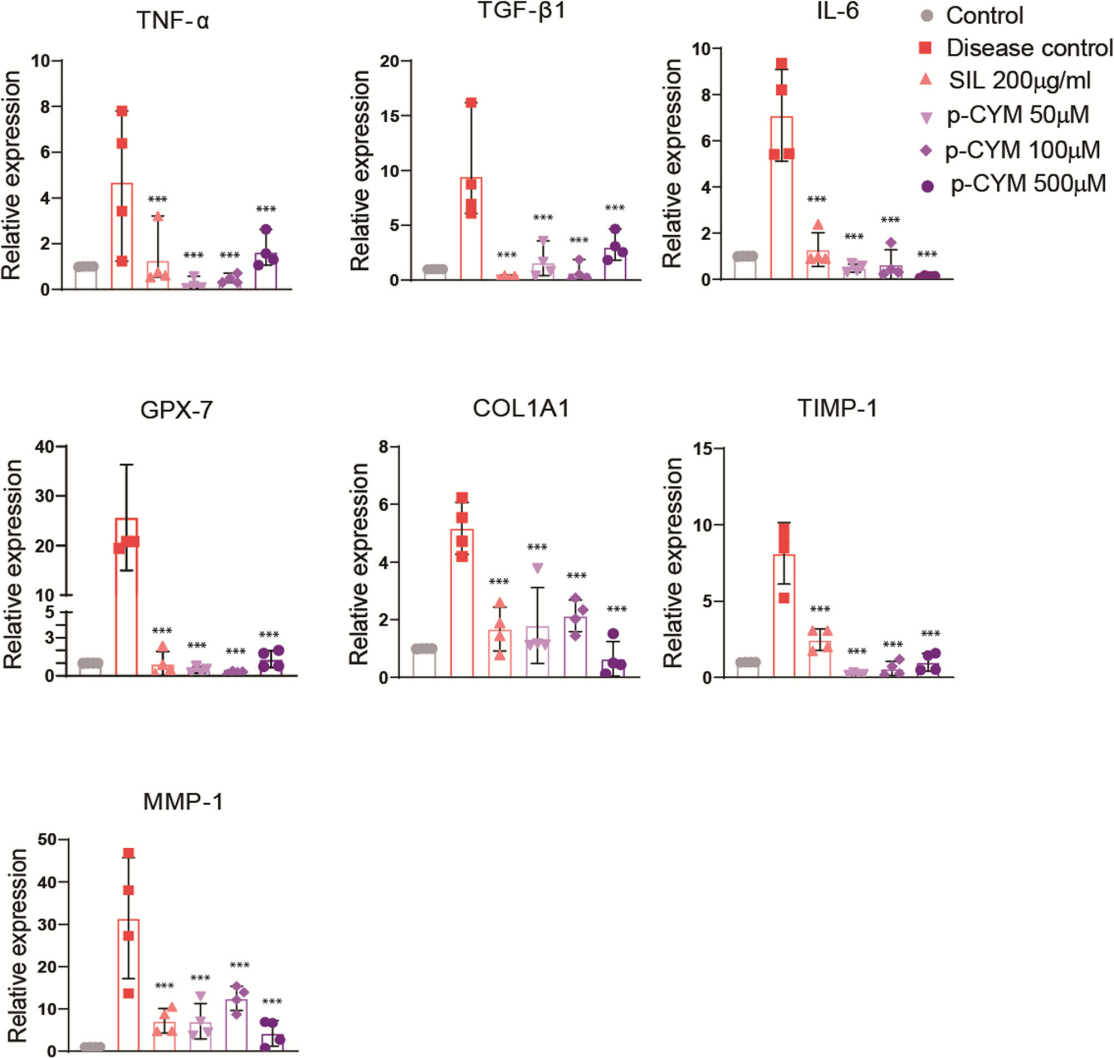
P-CYM displayed hepatoprotective effects by reducing transcript levels of *TNF-α*, *TGF-β1*, *IL-6*, *GPX-7*, *COL1A1*, *MMP-1,* and *TIMP-1*. *** ≤ 0.001 (treated groups vs disease group); one-way ANOVA followed by Tukey’s multiple comparison test; *n* = 3.

SOD and GSH activity increased in p-CYM- and SIL-treated groups compared to the disease group showing that p-CYM reduced oxidative stress induced by EtOH. Moreover, higher doses of p-CYM displayed more potent effects compared to SIL (Figure 3). The relative mRNA expression of biomarkers, including *TNF-α,* transforming growth factor-beta1 (*TGF-β1*), interleukin-6 (*IL-6*), glutathione peroxidase-7 (*GPX-7*), collagen type 1 (*COL1A1*)*, MMP-1,* and tissue inhibitor of MMP-1 (*TIMP-1*) were assessed to examine the molecular mechanism behind the hepatoprotective activity of p-CYM. The findings of this study showed that p-CYM significantly reduced the expression rate of the abovementioned biomarkers. These findings were equivalent to the standard drug “SIL.” In contrast to the treated groups, the expression rate of these biomarkers was higher in the disease group, which could be ascribed to toxicity induced by EtOH. The hepatoprotective role of p-CYM can be ascribed to the down-regulation of inflammatory and fibrotic markers (Figure 4).

### 3.6 P-CYM prevented CCl_4_-induced weight reduction and protected against CCl_4_-induced liver damage

One of the characteristics of chronic liver illness is weight loss, which may be caused by the liver’s metabolic dysfunction and a decrease in bile production, which in turn leads to a reduction in lipid emulsification and absorption [2,23]. The present findings also revealed that CCl_4_ reduced the body weight of rats, which was restored by treatment with SIL and p-CYM. Interestingly, the highest dose of p-CYM restored the body weight to normal levels, and this effect was much more prominent than in the SIL-treated group (Figure S4). Moreover, increased LFT levels reflect a variety of aberrant liver activities, including (a) hepatocellular instability, (b) decreased bile synthesis, and (c) altered protein synthesis, and are therefore indirect indicators of LF [2,23,24]. In current study, the exposure to CCl_4_ also led to increased levels of ALP, AST, ALT, and bilirubin, signaling liver damage. However, SIL and p-CYM treatment reduced these elevated markers, indicating their hepatoprotective effects (Figure S5).

### 3.7 Histopathological and real-time PCR analyses revealed anti-fibrotic effects of p-CYM

**Figure 5:**
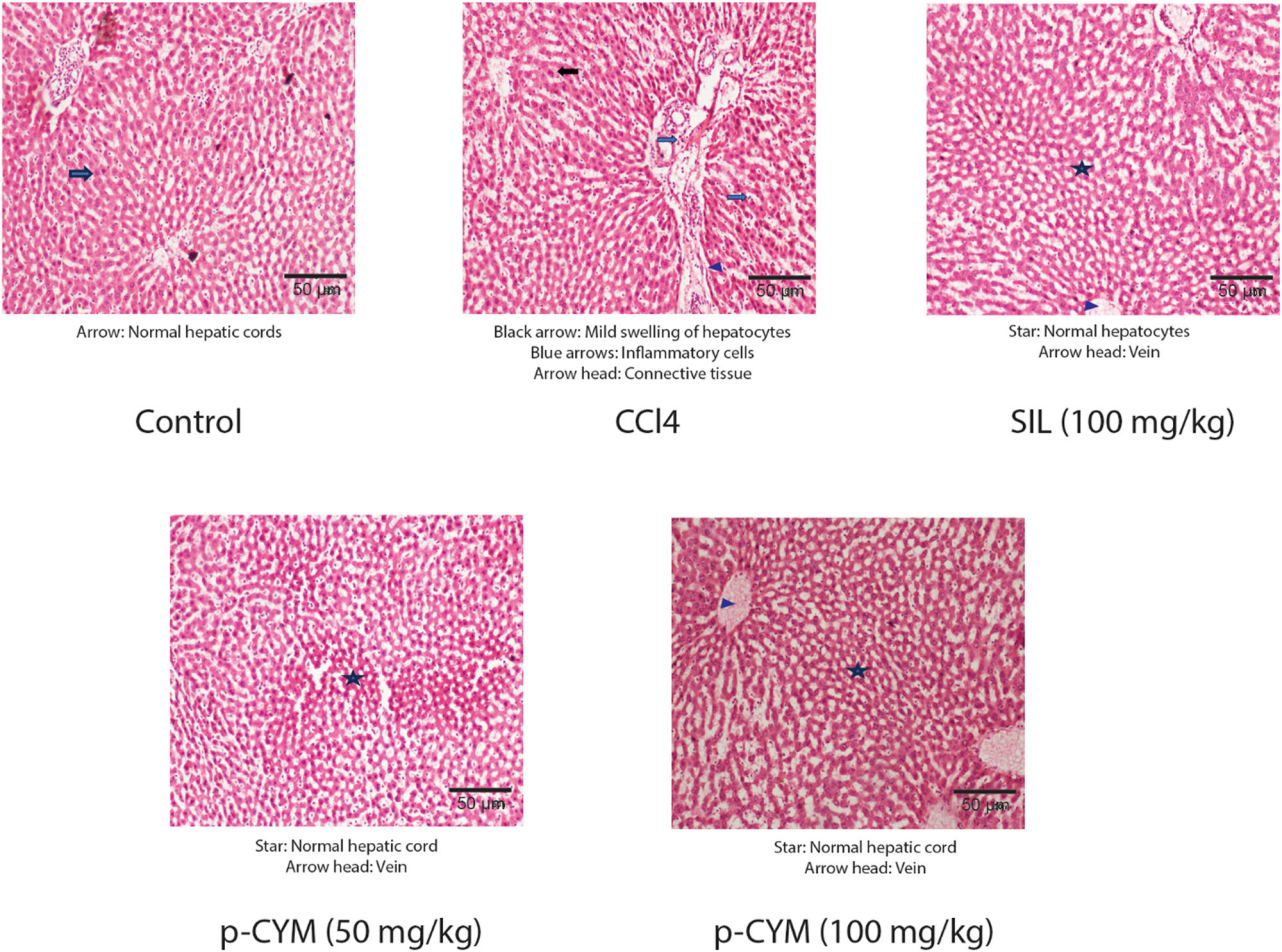
Histopathology of liver samples displayed anti-fibrotic effects of p-CYM.

**Figure 6:**
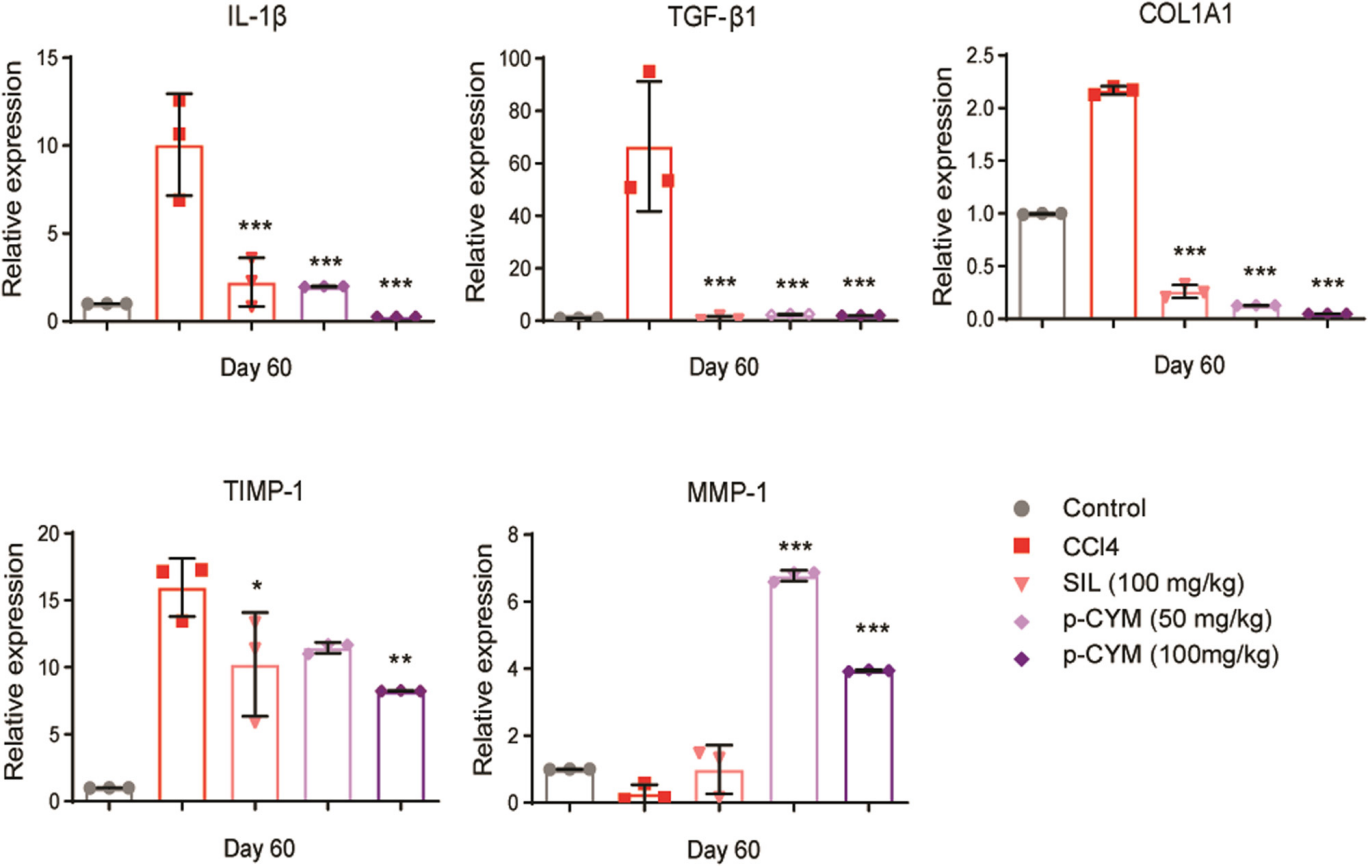
Modulation of pro- and anti-fibrotic markers by p-CYM. P-CYM and SIL reduced the transcript levels of *TIMP-1, IL-1β, COL1A1,* and *TGF-β1* and induced *MMP1,* indicating its anti-fibrotic effects. *** ≤ 0.001, ** ≤ 0.01, and * ≤ 0.05 (treated groups vs disease group); one-way ANOVA followed by Tukey’s multiple comparison test; *n* = 3.

The liver tissue samples of the disease group showed fibrotic scarring enriched with collagen and swollen hepatocytes. Treatment with SIL showed mild infiltration of inflammatory cells, while p-CYM (50 mg/kg)-treated tissue appeared normal with mild swelling of hepatocytes. At a higher dose (100 mg/kg), p-CYM did not display any inflammation or scarring, and the tissue also had a normal appearance. The above findings clearly indicated the protective effects of p-CYM against DEN–CCl_4_-induced LF (Figure 5, Table S2). RT-qPCR findings also demonstrated that CCl_4_ induced transcript levels of pro-fibrotic markers (*TIMP-1, IL-1β, COL1A1,* and *TGF-β1*) and reduced anti-fibrotic markers (*MMP-1*). Treatment with SIL and p-CYM restored these markers, indicating their anti-fibrotic effects (Figure 6).

### 3.8 P-CYM displayed strong binding affinity with TNF-α and modest affinity with MMP-1

**Figure 7:**
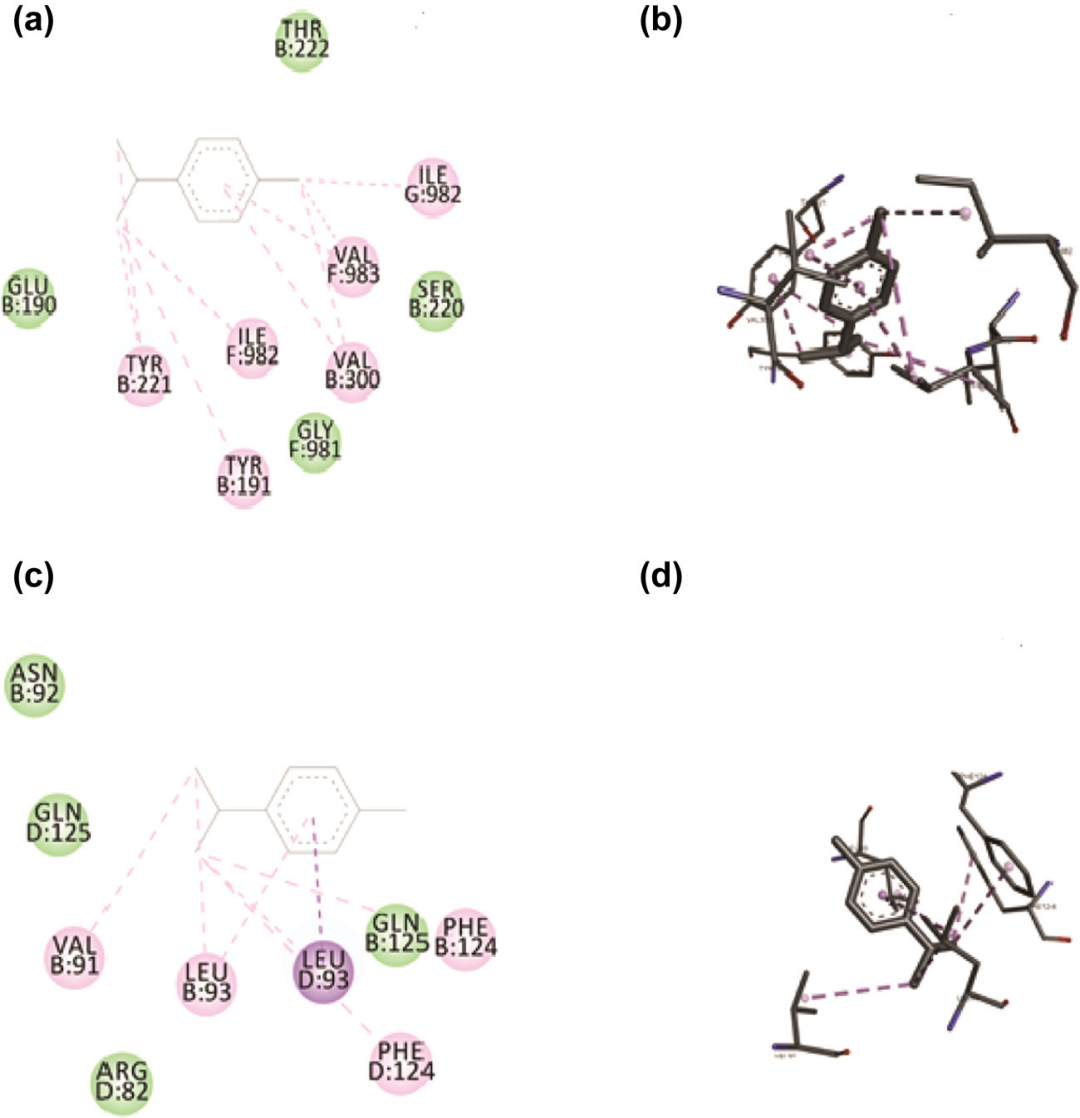
Binding interactions of p-CYM with TNF-α and MMP-1. 2D and 3D representation of binding interactions of p-CYM with the amino acid residues of the binding site of MMP-1 (a) and (b) and TNF-α (c) and (d).

The affinity between the ligands and protein targets was examined by molecular docking. The docking analysis was conducted using the AutoDock Vina [22] tool and PyRx [25] user interface. The best-docked posture complex and protein’s affinity were evaluated using the *E*-value (kcal/mol). It offered a prediction of the binding constant and free energy for docked ligands. Usually, if the binding energy is more negative, stronger is the interaction between ligand and target protein. P-CYM’s docking tests displayed strong binding interactions with TNF-α and MMP-1, and the results were in line with the pharmacological effects. The binding energies of p-CYM with TNF-α and MMP-1 were −6.1 and −5.4 kcal/mol, respectively. These findings indicate that p-CYM-induced anti-fibrotic effects could be due to direct interaction with TNF-α and MMP-1 (Figure 7).

## 4 Discussion

LF is a critical stage in the progression of chronic liver disease, occurring after the initial development of fatty liver disease. If left untreated, LF leads to significant liver damage, where the liver tissue undergoes structural changes, resulting in shrinkage and the formation of nodules, a condition known as cirrhosis. The progression from LF to cirrhosis and potentially to hepatocellular carcinoma (HCC) underscores the importance of targeting LF as a key therapeutic intervention to prevent further liver damage and mitigate the risk of life-threatening complications [26]. Despite its critical role in the progression of liver disease, LF remains a major clinical challenge due to the lack of effective treatments that can fully reverse or significantly alleviate fibrosis once it has developed. Currently, available therapies are limited in their ability to halt or reverse the fibrotic process, and no universally accepted treatment can cure or effectively manage LF on a long-term basis. This highlights the urgent need for the development of new, more effective therapies that can address LF at its core, prevent progression to cirrhosis, and reduce the risk of developing HCC.

In this study, we elucidated the cytoprotective ability of p-CYM against EtOH-induced injury in HepG2 cells and its anti-fibrotic potential against DEN–CCl_4_-induced LF in rats. Chronic consumption of EtOH accelerates liver damage, contributing to a range of liver diseases, such as cirrhosis, HCC, alcoholic hepatitis, and alcoholic steatosis [27,28]. High levels of EtOH intake induce oxidative stress and fat accumulation in hepatocytes, leading to liver injury. Reactive oxygen species (ROS) play a central role in oxidative stress-induced cell death [29,30]. Studies have shown that excessive ROS production in the liver causes abnormal protein expression, oxidative DNA damage, and disruption of cell membranes, worsening liver function [31,32]. To prevent the progression of alcoholic liver disease (ALD), reducing EtOH-induced oxidative stress and fat buildup in the liver could be beneficial [33]. In addition to EtOH, DEN and CCl_4_ are commonly used in animal models to induce LF. In both wild-type and transgenic animal models, administration of DEN and CCl_4_ for 3–4 weeks causes centrilobular and periportal LF. CCl_4_ specifically activates macrophages and hepatic stellate cells (HSCs), triggering the upregulation of pro-fibrotic mediators and increasing ROS generation. This leads to fibrogenesis and cirrhosis, primarily driven by the overproduction of TGF-β [26]. The key features of DEN–CCl_4_-induced LF include weight loss, increased LFTs, and fibrotic changes in the liver [23,24]. These findings highlight the importance of targeting oxidative stress and fibrotic pathways to prevent or treat ALD and associated liver damage.

Moreover, studies have shown that anti-oxidants alter the redox state of the cell and reduce the generation of free radicals. Some of these agents include vitamins E and C, N-acetylcysteine (NAC), mitoquinone, and polyenylphosphatidylcholine. Vitamin E stabilizes the free radical compounds by forming complexes with the unpaired electrons and prevents the activation of HSCs [34]. In a small open-label study, vitamin E treatment (1200 IU/day) for 8 weeks stopped the fibrogenesis cascade in six patients who were refractory to interferon. This was demonstrated by decreased levels of malonaldehyde and reduced activation of HSCs [34]. Similarly, a moderate reduction in serum ALT to 63 IU/l from baseline levels of 73 IU/l was observed in 17 patients who received vitamin E treatment (500 mg/day) for three months [35]. Previous studies report that humans experience severe functional and structural alterations after ingesting EtOH, which disrupts metabolism. GSH and SOD depletion occurs in case of EtOH exposure owing to their rapid utilization by ROS [36,37].

In our study, several assays also suggested that pre-treatment of HepG2 cells with p-CYM significantly reduced EtOH-induced oxidative stress and cell death. The present findings indicated that pre-treatment with p-CYM prevented the decrease in GSH and SOD levels in HepG2 cells. The restoration of the intracellular GSH and SOD levels in HepG2 cells pre-exposed with p-CYM indicated that increased amounts of EtOH-induced ROS could be scavenged, which subsequently resulted in reduced cellular damage. Furthermore, the pathological changes associated with DEN–CCl_4_-induced LF were significantly reduced by p-CYM, illustrating its beneficial effects in the resolution of LF.

In order to elucidate the possible molecular mechanism behind the hepatoprotective effects of p-CYM, we assessed the transcription levels of several pro- and anti-fibrotic biomarkers. Studies have revealed that pro-inflammatory mediators are crucial to the process of fibrogenesis, and those who suffer from chronic liver conditions have elevated IL-1β levels in their serum [38,39]. IL-1β is currently regarded as a key regulator of tissue injury and inflammation in chronic liver disorders because of its critical role in the transformation of steatosis into steatohepatitis and LF [40,41,42]. Our tested compound, p-CYM, showed potential anti-fibrotic properties by significantly reducing mRNA expression of *IL-1β* in rats.

Other cytokine family members that promote both acute and chronic inflammation in the liver include TNF-α and IL-6 [43,44,45]. The IKK and JNK pathways are activated by TNF receptor interactions by bringing in the adaptor molecules [46]. IKK phosphorylates IκB and p65, resulting in NF-κB activation [47]. Increased JNK activity tips the scales in favor of cell death by causing the E3 ligase to be phosphorylated, followed by the ubiquitination and degradation of the NF-κB-regulated caspase-8 inhibitor “c-Flip.” Prolonged activation of JNK necessitates TNF-α-induced ROS generation and moves the balance toward cell death [48]. Moreover, liver inflammation caused by EtOH is believed to be exacerbated by the pro-inflammatory cytokine IL-6, which plays a critical role in the progression of liver damage [49,50,51]. Elevated levels of IL-6 have been linked to the activation of several downstream signaling pathways, including the Janus kinase (JAK)/signal transducer and activator of the transcription 3 (STAT3) pathway. This pathway is a key mediator of LF and contributes to the chronic inflammatory environment associated with ALD. The activation of JAK/STAT3 by IL-6 leads to the transcription of target genes involved in fibrosis, inflammation, and cell survival, promoting the persistence of HSC activation and ECM deposition. This cascade not only worsens liver inflammation but also accelerates the progression of fibrosis, thereby playing a pivotal role in the pathogenesis of EtOH-induced liver injury [52]. Our study also revealed an increased expression rate of these biomarkers in EtOH-intoxicated HepG2 cells, and p-CYM effectively reduced the transcript levels of *TNF-α* and *IL-6,* reiterating its protective mechanism.

TGF-β1 is another significant regulator of liver cell growth and plays a role in the progression of chronic liver damage [53]. Numerous studies have shown that EtOH-induced inflammation results in the production of TGF-β1, which is thought to be crucial for the pathophysiology and development of ALD [54]. One of the primary proteins that promotes fibrogenesis is TGF-β1, which stimulates HSC and causes them to activate and produce ECM proteins [55]. Previous studies have indicated that LF is associated with high levels of TGF-β1 and COL1A1 expression, and the collagen deposition increases as the fibrosis progresses [56,57,58,59]. In the present study, we also witnessed increased levels of *TGF-β1* and *COL1A1* in EtOH- and DEN–CCl_4_-induced hepatic damage, while p-CYM significantly reduced these biomarkers, illustrating its anti-fibrotic effect.

TIMP and MMP proteins normally exist in equilibrium in healthy tissue; however, due to chronic liver damage, TIMP-1 levels increase than MMP-1 levels, which results in inhibition of ECM breakdown [60,61]. Advanced stages of LF include almost six times the normal amount of ECM, which includes proteoglycans, fibronectin, elastin, laminin, hyaluronan, and collagens I, III, and IV. Both increased synthesis and decreased degradation lead to the accumulation of ECM proteins [62]. The primary cause of the decreased activity of ECM-removing MMPs is the overexpression of their particular inhibitors (TIMPs). The breakdown of ECM proteins and programmed cell death of HSCs are the mechanisms underlying the MMP-derived inhibition of the fibrogenic response [63,64]. In this study, we also witnessed a reduction in *TIMP-1* and induction in *MMP-1* levels upon treatment with p-CYM, indicating that p-CYM has the ability to reduce fibrogenic response by promoting ECM degradation.

Overall, this study highlights the therapeutic potential of p-CYM against EtOH- and CCl₄-induced hepatotoxicity, suggesting its viability as a protective agent against liver damage. Despite its promise, one key limitation of p-CYM, like many natural compounds, may be its relatively low bioavailability, which can restrict its therapeutic efficacy. To address this challenge, future research could focus on developing a nanoformulation of p-CYM.

Nanoformulations have emerged as a cutting-edge approach in drug delivery, particularly for compounds that face challenges related to solubility, stability, or targeted delivery. By incorporating p-CYM into nanocarriers, its bioavailability can be significantly improved, ensuring more effective systemic circulation and cellular uptake. Moreover, nanomaterials offer several distinct advantages that make them highly suitable for this purpose. These include their large drug-loading capacity, which allows for the encapsulation of significant amounts of therapeutic agents, and their surface modification capabilities, enabling the development of targeted delivery systems. Such targeted systems could direct p-CYM specifically to the liver, minimizing off-target effects and enhancing therapeutic precision. Additionally, nanocarriers can offer controlled release profiles, ensuring sustained therapeutic levels of the drug over time [65,66,67].

Given the substantial progress in the field of nanotechnology, this approach could transform p-CYM from a promising natural compound into a highly effective therapeutic agent. Future investigations could explore various nanocarrier systems, such as liposomes, polymeric nanoparticles, or lipid-based nanocarriers, to identify the most suitable platform for p-CYM delivery. Collectively, these advancements could pave the way for the clinical translation of p-CYM as a novel hepatoprotective therapy.

## 5 Conclusion

Based on *in vitro* findings, it can be concluded from this study that EtOH intoxication in the HepG2 cell line induces oxidative stress, inflammation, and collagen synthesis, as evident by reduced anti-oxidants (GSH and SOD) and increased levels of inflammatory (*IL-1β* and *TNF-α*) and pro-fibrotic (*TGF-β1* and *COL1A1*) biomarkers. P-CYM protected HepG2 cells from EtOH-induced cell death owing to its anti-oxidant and anti-inflammatory properties. Moreover, p-CYM also reduced the levels of pro-fibrotic mediators under *in vitro* settings. Similarly, under *in vivo* conditions, DEN- CCl_4_ induced oxidative stress and fibrosis, while treatment with p-CYM effectively reversed pro-fibrotic effects of DEN–CCl_4_. In a nutshell, it is conceivable from this study that the hepatoprotective effects of p-CYM could be attributed to its anti-oxidant, anti-inflammatory, and ECM modulatory activities.

## Supplementary Material

Supplementary material

## References

[1] Kisseleva T, Brenner D. Molecular and cellular mechanisms of liver fibrosis and its regression. Nat Rev Gastroenterol Hepatol. 2021;18(3):151–66. 10.1038/s41575-020-00372-7 33128017

[2] Benitez R, Caro M, Andres‐Leon E, O’Valle F, Delgado M. Cortistatin regulates fibrosis and myofibroblast activation in experimental hepatotoxic‐and cholestatic‐induced liver injury. Br J Pharmacol. 2022;179(10):2275–96. 10.1111/bph.15752 34821378

[3] Yoo ER, Cholankeril G, Ahmed A. Treating alcohol use disorder in chronic liver disease. Clin Liver Dis (Hoboken). 2020;15(2):77–80. 10.1002/cld.881 PMC7098671 32226621

[4] Stashin AR, Fikse DJ, Orta AM, Briggs 3rd RP, Wheatley SM, Koons AL. You dropped the bomb on me: A case series of carbon tetrachloride toxicity. Cureus. 2023;15(4):e37879. 10.7759/cureus.37879 PMC10202662 37223155

[5] Dey D, Chaskar S, Bhatt N, Chitre D. Hepatoprotective activity of BV-7310, a Proprietary herbal formulation of Phyllanthus niruri, Tephrosia purpurea, Boerhavia diffusa, and Andrographis paniculata, in alcohol-induced HepG2 cells and alcohol plus a haloalkane, CCl(4), induced liver damage in rats. Evid Based Complement Alternat Med. 2020;2020:6428906. 10.1155/2020/6428906 PMC7132358 32308713

[6] Manzo-Avalos S, Saavedra-Molina A. Cellular and mitochondrial effects of alcohol - consumption. Int J Environ Res Public Health. 2010;7(12):4281–304. 10.3390/ijerph7124281 PMC3037055 21318009

[7] Huang W, Booth DM, Cane MC, Chvanov M, Javed MA, Elliott VL, et al. Fatty acid ethyl ester synthase inhibition ameliorates ethanol-induced Ca2+ -dependent mitochondrial dysfunction and acute pancreatitis. Gut. 2014;63(8):1313–24. 10.1136/gutjnl-2012-304058 PMC4112447 24162590

[8] Lee KJ, Choi JH, Jeong HG. Hepatoprotective and antioxidant effects of the coffee diterpenes kahweol and cafestol on carbon tetrachloride-induced liver damage in mice. Food Chem Toxicol. 2007;45(11):2118–25. 10.1016/j.fct.2007.05.010 17590492

[9] Tilg H, Day CP. Management strategies in alcoholic liver disease. Nat Clin Pract Gastroenterol Hepatol. 2007;4(1):24–34. 10.1038/ncpgasthep0683 17203086

[10] Muriel P, Rivera-Espinoza Y. Beneficial drugs for liver diseases. J Appl Toxicol. 2008;28(2):93–103. 10.1002/jat.1310 17966118

[11] Stickel F, Schuppan D. Herbal medicine in the treatment of liver diseases. Dig Liver Dis. 2007;39(4):293–304. 10.1016/j.dld.2006.11.004 17331820

[12] Osna NA, Donohue Jr TM, Kharbanda KK. Alcoholic liver disease: Pathogenesis and current management. Alcohol Res. 2017;38(2):147–61. 10.35946/arcr.v38.2.01 PMC5513682 28988570

[13] Abe R, Ohtani K. An ethnobotanical study of medicinal plants and traditional therapies on Batan Island, the Philippines. J Ethnopharmacol. 2013;145(2):554–65. 10.1016/j.jep.2012.11.029 23183086

[14] Atanasov AG, Waltenberger B, Pferschy-Wenzig EM, Linder T, Wawrosch C, Uhrin P, et al. Discovery and resupply of pharmacologically active plant-derived natural products: A review. Biotechnol Adv. 2015;33(8):1582–614. 10.1016/j.biotechadv.2015.08.001 PMC4748402 26281720

[15] Balahbib A, El Omari N, Hachlafi NE, Lakhdar F, El Menyiy N, Salhi N, et al. Health beneficial and pharmacological properties of p-cymene. Food Chem Toxicol. 2021;153:112259. 10.1016/j.fct.2021.112259 33984423

[16] Shareef SH, Al-Medhtiy MH, Ibrahim IAA, Alzahrani AR, Jabbar AA, Galali Y, et al. Gastroprophylactic effects of p-cymene in ethanol-induced gastric ulcer in rats. Processes. 2022;10(7):1314.

[17] Wang S, Wang X, Wang YU, Leng Q, Sun YU, Hoffman RM, et al. The anti-oxidant monoterpene p-cymene reduced the occurrence of colorectal cancer in a hyperlipidemia rat model by reducing oxidative stress and expression of inflammatory cytokines. Anticancer Res. 2021;41(3):1213–8. 10.21873/anticanres.14878 33788712

[18] Maqbool T, Awan SJ, Malik S, Hadi F, Shehzadi S, Tariq K. In-vitro anti-proliferative, apoptotic and antioxidative activities of medicinal herb Kalonji (Nigella sativa). Curr Pharm Biotechnol. 2019;20(15):1288–308. 10.2174/1389201020666190821144633 31433749

[19] Lee JY, Kim H, Jeong Y, Kang CH. Lactic acid bacteria exert a hepatoprotective effect against ethanol-induced liver injury in HepG2 cells. Microorganisms. 2021;9(9):1844. 10.3390/microorganisms9091844 PMC8465258 34576738

[20] Pouresmaeil V, Al Abudi AH, Mahimid AH, Sarafraz Yazdi M, Es-Haghi A. Evaluation of serum selenium and copper levels with inflammatory cytokines and indices of oxidative stress in type 2 diabetes. Biol Trace Elem Res. 2023;201(2):617–26. 10.1007/s12011-022-03191-w 35279796

[21] Willard L, Ranjan A, Zhang H, Monzavi H, Boyko RF, Sykes BD, et al. VADAR: A web server for quantitative evaluation of protein structure quality. Nucleic Acids Res. 2003;31(13):3316–9. 10.1093/nar/gkg565 PMC168972 12824316

[22] Trott O, Olson AJ. AutoDock Vina: Improving the speed and accuracy of docking with a new scoring function, efficient optimization, and multithreading. J Comput Chem. 2010;31(2):455–61. 10.1002/jcc.21334 PMC3041641 19499576

[23] Brattin WJ, Glende Jr EA, Recknagel RO. Pathological mechanisms in carbon tetrachloride hepatotoxicity. J Free Radic Biol Med. 1985;1(1):27–38. 10.1016/0748-5514(85)90026-1 3915301

[24] Nadkarni GD, D’Souza NB. Hepatic antioxidant enzymes and lipid peroxidation in carbon tetrachloride-induced liver cirrhosis in rats. Biochem Med Metab Biol. 1988;40(1):42–5. 10.1016/0885-4505(88)90102-8 3219229

[25] Dallakyan S, Olson AJ. Small-molecule library screening by docking with PyRx. In: Hempel JE. Chemical biology: Methods and protocols. New York, USA: Springer; 2015. pp. 243–50. 10.1007/978-1-4939-2269-7_19 25618350

[26] Gao HY, Li GY, Lou MM, Li XY, Wei XY, Wang JH. Hepatoprotective effect of matrine salvianolic acid B salt on carbon tetrachloride-induced hepatic fibrosis. J Inflamm (Lond). 2012;9(1):16. 10.1186/1476-9255-9-16 PMC3404020 22559721

[27] Gao B, Bataller R. Alcoholic liver disease: Pathogenesis and new therapeutic targets. Gastroenterology. 2011;141(5):1572–85. 10.1053/j.gastro.2011.09.002 PMC3214974 21920463

[28] Orman ES, Odena G, Bataller R. Alcoholic liver disease: Pathogenesis, management, and novel targets for therapy. J Gastroenterol Hepatol. 2013;28 Suppl 1(S1):77–84. 10.1111/jgh.12030 PMC4405238 23855300

[29] Chen WM, Shaw LH, Chang PJ, Tung SY, Chang TS, Shen CH, et al. Hepatoprotective effect of resveratrol against ethanol-induced oxidative stress through induction of superoxide dismutase in vivo and in vitro. Exp Ther Med. 2016;11(4):1231–8. 10.3892/etm.2016.3077 PMC4812565 27073428

[30] Kannan K, Jain SK. Oxidative stress and apoptosis. Pathophysiology. 2000;7(3):153–63. 10.1016/s0928-4680(00)00053-5 10996508

[31] Chen Z, Tian R, She Z, Cai J, Li H. Role of oxidative stress in the pathogenesis of nonalcoholic fatty liver disease. Free Radic Biol Med. 2020;152:116–41. 10.1016/j.freeradbiomed.2020.02.025 32156524

[32] Machida K, Cheng KT, Lai CK, Jeng KS, Sung VM, Lai MM. Hepatitis C virus triggers mitochondrial permeability transition with production of reactive oxygen species, leading to DNA damage and STAT3 activation. J Virol. 2006;80(14):7199–207. 10.1128/JVI.00321-06 PMC1489016 16809325

[33] Nagappan A, Jung DY, Kim J-H, Lee H, Jung MH. Gomisin N alleviates ethanol-induced liver injury through ameliorating lipid metabolism and oxidative stress. Int J Mol Sci. 2018;19(9):2601. 10.3390/ijms19092601 PMC6164513 30200508

[34] Houglum K, Venkataramani A, Lyche K, Chojkier M. A pilot study of the effects of d-alpha-tocopherol on hepatic stellate cell activation in chronic hepatitis C. Gastroenterology. 1997;113(4):1069–73. 10.1053/gast.1997.v113.pm9322499 9322499

[35] Mahmood S, Yamada G, Niiyama G, Kawanaka M, Togawa K, Sho M, et al. Effect of vitamin E on serum aminotransferase and thioredoxin levels in patients with viral hepatitis C. Free Radic Res. 2003;37(7):781–5. 10.1080/1071576031000102141 12911275

[36] Bae D, You Y, Yoon H-G, Kim K, Lee Y-H, Kim Y, et al. Protective effects of loquat (Eriobotrya japonica) leaves against ethanol-induced toxicity in HepG2 cells transfected with CYP2E1. Food Sci Biotechnol. 2010;19:1093–6.

[37] Hartley‐Whitaker J, Ainsworth G, Meharg AA. Copper‐and arsenate‐induced oxidative stress in Holcus lanatus L. clones with differential sensitivity. Plant Cell Eniron. 2001;24(7):713–22.

[38] Ludwiczek O, Vannier E, Moschen A, Salazar-Montes A, Borggraefe I, Gabay C, et al. Impaired counter-regulation of interleukin-1 by the soluble IL-1 receptor type II in patients with chronic liver disease. Scand J Gastroenterol. 2008;43(11):1360–5. 10.1080/00365520802179925 18609176

[39] Tilg H, Vogel W, Wiedermann CJ, Shapiro L, Herold M, Judmaier G, et al. Circulating interleukin-1 and tumor necrosis factor antagonists in liver disease. Hepatology. 1993;18(5):1132–8. 8225219

[40] Kamari Y, Shaish A, Vax E, Shemesh S, Kandel-Kfir M, Arbel Y, et al. Lack of interleukin-1alpha or interleukin-1beta inhibits transformation of steatosis to steatohepatitis and liver fibrosis in hypercholesterolemic mice. J Hepatol. 2011;55(5):1086–94. 10.1016/j.jhep.2011.01.048 PMC3210940 21354232

[41] Tilg H, Moschen AR, Szabo G. Interleukin-1 and inflammasomes in alcoholic liver disease/acute alcoholic hepatitis and nonalcoholic fatty liver disease/nonalcoholic steatohepatitis. Hepatology. 2016;64(3):955–65. 10.1002/hep.28456 26773297

[42] Gieling RG, Wallace K, Han YP. Interleukin-1 participates in the progression from liver injury to fibrosis. Am J Physiol Gastrointest Liver Physiol. 2009;296(6):G1324–31. 10.1152/ajpgi.90564.2008 PMC2697947 19342509

[43] Shaikh PZ. Cytokines & their physiologic and pharmacologic functions in inflammation: A review. Int J Pharm Life Sci. 2011;2(11):1247–63.

[44] Feghali CA, Wright TM. Cytokines in acute and chronic inflammation. Front Biosci. 1997;2(4):d12–26. 10.2741/a171 9159205

[45] Holtmann MH, Neurath MF. Differential TNF-signaling in chronic inflammatory disorders. Curr Mol Med. 2004;4(4):439–44. 10.2174/1566524043360636 15354874

[46] Baud V, Karin M. Signal transduction by tumor necrosis factor and its relatives. Trends Cell Biol. 2001;11(9):372–7. 10.1016/s0962-8924(01)02064-5 11514191

[47] Schwabe RF, Sakurai H. IKKβ phosphorylates p65 at S468 in transactivaton domain 2. FASEB J. 2005;19(12):1758–60. 10.1096/fj.05-3736fje 16046471

[48] Kamata H, Honda S, Maeda S, Chang L, Hirata H, Karin M. Reactive oxygen species promote TNFalpha-induced death and sustained JNK activation by inhibiting MAP kinase phosphatases. Cell. 2005;120(5):649–61. 10.1016/j.cell.2004.12.041 15766528

[49] Gao B. Cytokines, STATs and liver disease. Cell Mol Immunol. 2005;2(2):92–100. 16191414

[50] Mitra SK, Varma SR, Godavarthi A, Nandakumar KS. Liv.52 regulates ethanol induced PPARgamma and TNF alpha expression in HepG2 cells. Mol Cell Biochem. 2008;315(1–2):9–15. 10.1007/s11010-008-9782-9 18449625

[51] Kawaratani H, Tsujimoto T, Douhara A, Takaya H, Moriya K, Namisaki T, et al. The effect of inflammatory cytokines in alcoholic liver disease. Mediators Inflamm. 2013;2013:495156. 10.1155/2013/495156 PMC3872233 24385684

[52] Li Y, Zhao J, Yin Y, Li K, Zhang C, Zheng Y. The role of IL-6 in fibrotic Diseases: Molecular and cellular mechanisms. Int J Biol Sci. 2022;18(14):5405–14. 10.7150/ijbs.75876 PMC9461670 36147459

[53] Dooley S, ten Dijke P. TGF-beta in progression of liver disease. Cell Tissue Res. 2012;347(1):245–56. 10.1007/s00441-011-1246-y PMC3250614 22006249

[54] Song Z, Deaciuc I, Song M, Lee DY, Liu Y, Ji X, et al. Silymarin protects against acute ethanol-induced hepatotoxicity in mice. Alcohol Clin Exp Res. 2006;30(3):407–13. 10.1111/j.1530-0277.2006.00063.x PMC4217313 16499481

[55] Dewidar B, Soukupova J, Fabregat I, Dooley S. TGF-β in hepatic stellate cell activation and liver fibrogenesis: Updated. Curr Pathobiol Rep. 2015;3(4):291–305.

[56] Badylak SF, Freytes DO, Gilbert TW. Extracellular matrix as a biological scaffold material: Structure and function. Acta Biomater. 2009;5(1):1–13. 10.1016/j.actbio.2008.09.013 18938117

[57] Karsdal MA, Nielsen SH, Leeming D, Langholm L, Nielsen M, Manon-Jensen T, et al. The good and the bad collagens of fibrosis–their role in signaling and organ function. Adv Drug Deliv Rev. 2017;121:43–56. 10.1016/j.addr.2017.07.014 28736303

[58] Liu X, Hu H, Yin JQ. Therapeutic strategies against TGF-beta signaling pathway in hepatic fibrosis. Liver Int. 2006;26(1):8–22. 10.1111/j.1478-3231.2005.01192.x 16420505

[59] Liu M, Xu Y, Han X, Yin L, Xu L, Qi Y, et al. Dioscin alleviates alcoholic liver fibrosis by attenuating hepatic stellate cell activation via the TLR4/MyD88/NF-kappaB signaling pathway. Sci Rep. 2015;5(1):18038. 10.1038/srep18038 PMC4674875 26655640

[60] Hemmann S, Graf J, Roderfeld M, Roeb E. Expression of MMPs and TIMPs in liver fibrosis - a systematic review with special emphasis on anti-fibrotic strategies. J Hepatol. 2007;46(5):955–75. 10.1016/j.jhep.2007.02.003 17383048

[61] Roeb E, Purucker E, Breuer B, Nguyen H, Heinrich PC, Rose-John S, et al. TIMP expression in toxic and cholestatic liver injury in rat. J Hepatol. 1997;27(3):535–44. 10.1016/s0168-8278(97)80359-5 9314132

[62] Robert S, Gicquel T, Bodin A, Lagente V, Boichot E. Characterization of the MMP/TIMP imbalance and collagen production induced by IL-1beta or TNF-alpha release from human hepatic stellate cells. PLoS One. 2016;11(4):e0153118. 10.1371/journal.pone.0153118 PMC4821480 27046197

[63] Murphy FR, Issa R, Zhou X, Ratnarajah S, Arthur MJ, Benyon C, et al. Inhibition of apoptosis of activated hepatic stellate cells by tissue inhibitor of metalloproteinase-1 is mediated via effects on matrix metalloproteinase inhibition: Implications for reversibility of liver fibrosis. J Biol Chem. 2002;277(13):11069–76. 10.1074/jbc.M111490200 11796725

[64] Iimuro Y, Brenner DA. Matrix metalloproteinase gene delivery for liver fibrosis. Pharm Res. 2008;25(2):249–58. 10.1007/s11095-007-9311-7 PMC2245995 17577645

[65] Zhu X, Li S. Nanomaterials in tumor immunotherapy: New strategies and challenges. Mol Cancer. 2023;22(1):94. 10.1186/s12943-023-01797-9 PMC10262535 37312116

[66] Kang Y, Li S. Nanomaterials: Breaking through the bottleneck of tumor immunotherapy. Int J Biol Macromol. 2023;230:123159. 10.1016/j.ijbiomac.2023.123159 36610572

[67] Chen Z, Yue Z, Yang K, Li S. Nanomaterials: Small particles show huge possibilities for cancer immunotherapy. J Nanobiotechnology. 2022;20(1):484. 10.1186/s12951-022-01692-3 PMC9668401 36384524

