# Supplementary material for: p-Cymene inhibits pro-fibrotic and inflammatory mediators to prevent hepatic dysfunction
Source: Open Life Sci. 2025 Apr 15;20(1):20221054. doi: 10.1515/biol-2022-1054 (PMC12032992; doi:10.1515/biol-2022-1054)
Supplement: Supplementary material [file biol-2022-1054-sm.pdf]

Supplementary material

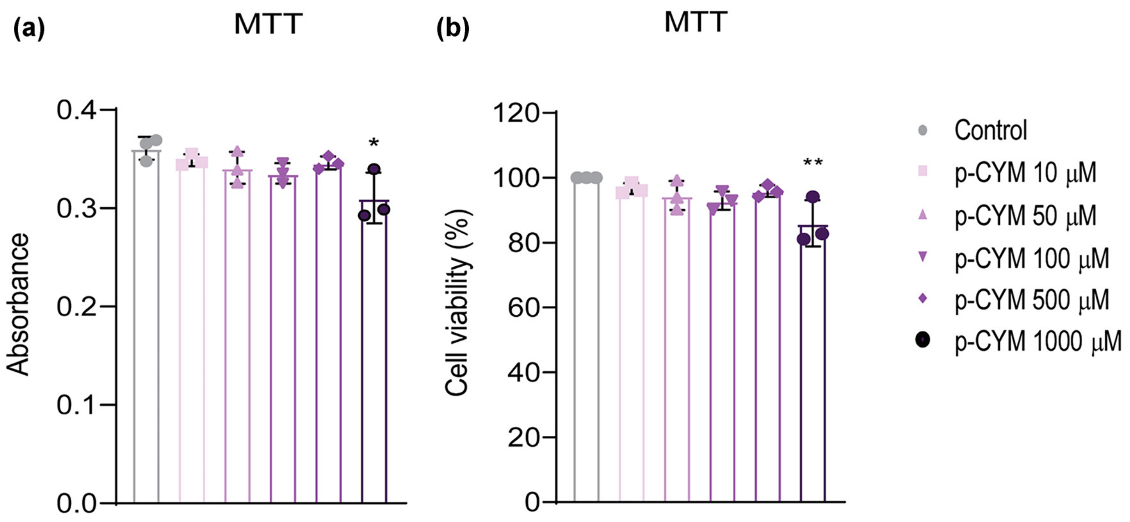

Figure S1: p-CYM did not reduce cell viability except at 1,000 μM. MTT assay showing cell viability at different concentrations (10, 50, 100, 500 and 1,000 μM) of p-CYM. At 1,000 μM concenteration, p-CYM significantly reduced cell proliferation. **≤0.01, *≤0.05 (One-way ANOVA followed by Tukey's multiple comparison test; *n* = 3).

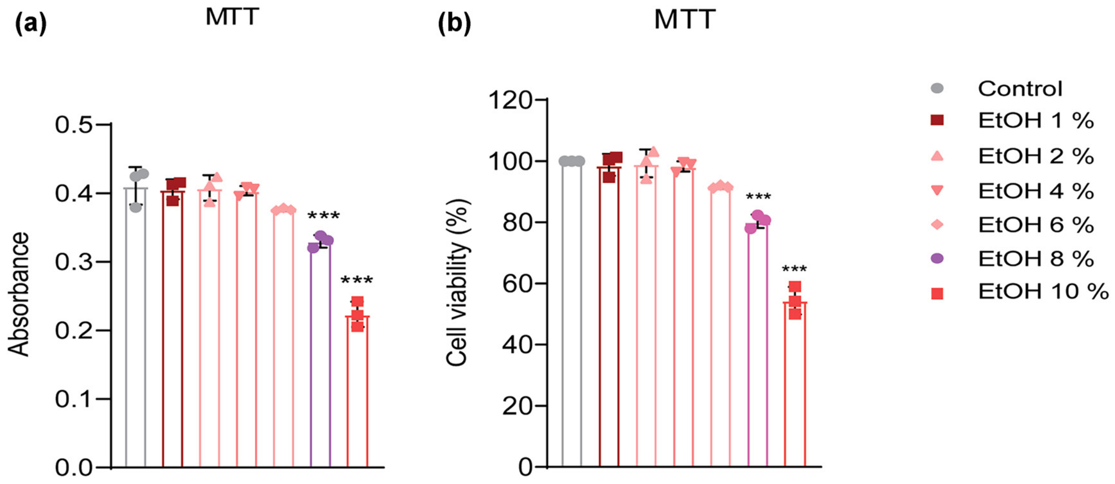

Figure S2: EtOH treatment demonstrated cytotoxicity at higher doses. HepG2 cells were subjected to increasing concentrations of EtOH for 24 h. EtOH significantly induced toxicity at 8 and 10% concentrations as witnessed by reduction in cell viability . ***≤0.001 (One-way ANOVA followed by Tukey's multiple comparison test; *n* = 3).

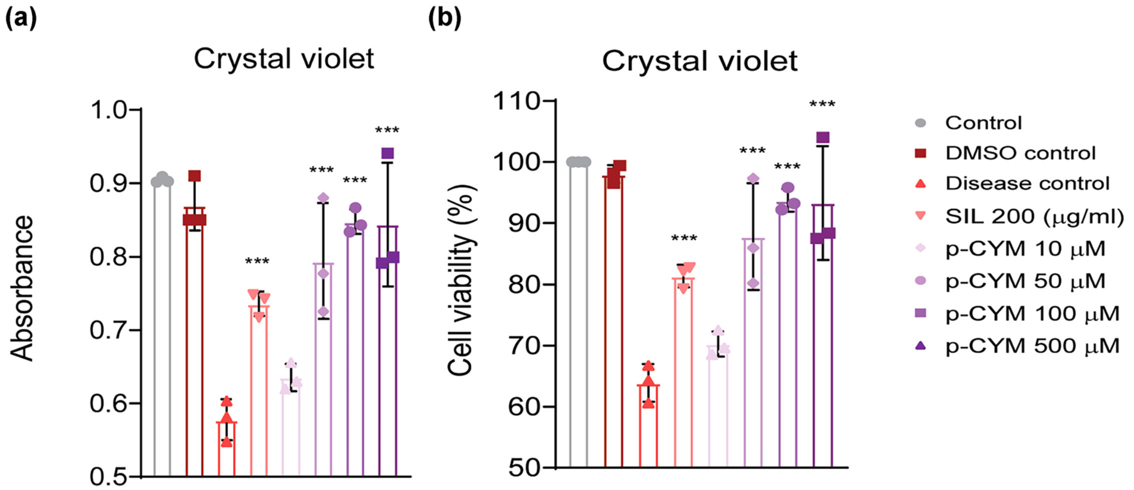

Figure S3: p-CYM prevented cytotoxic effects of EtOH in HepG2 cells. Ethanol treatment reduced viable cell count as compared to control group. Pre-treatment with p-CYM and SIL protected against EtOH toxicity in HepG2 cells. ***≤0.001 (One-way ANOVA followed by Tukey's multiple comparison test; *n* = 3).

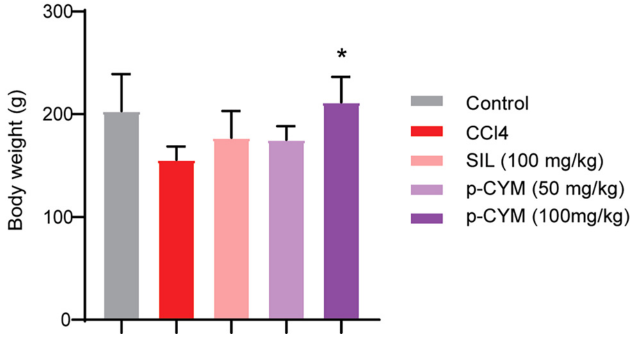

Figure S4: p-CYM prevented CCl4 induced weight loss. Weight reduction was observed in disease group while p-CYM and SIL treated groups showed a marked improvement in body weight. *≤0.05 (One-way ANOVA followed by Tukey's multiple comparison test; *n* = 4).

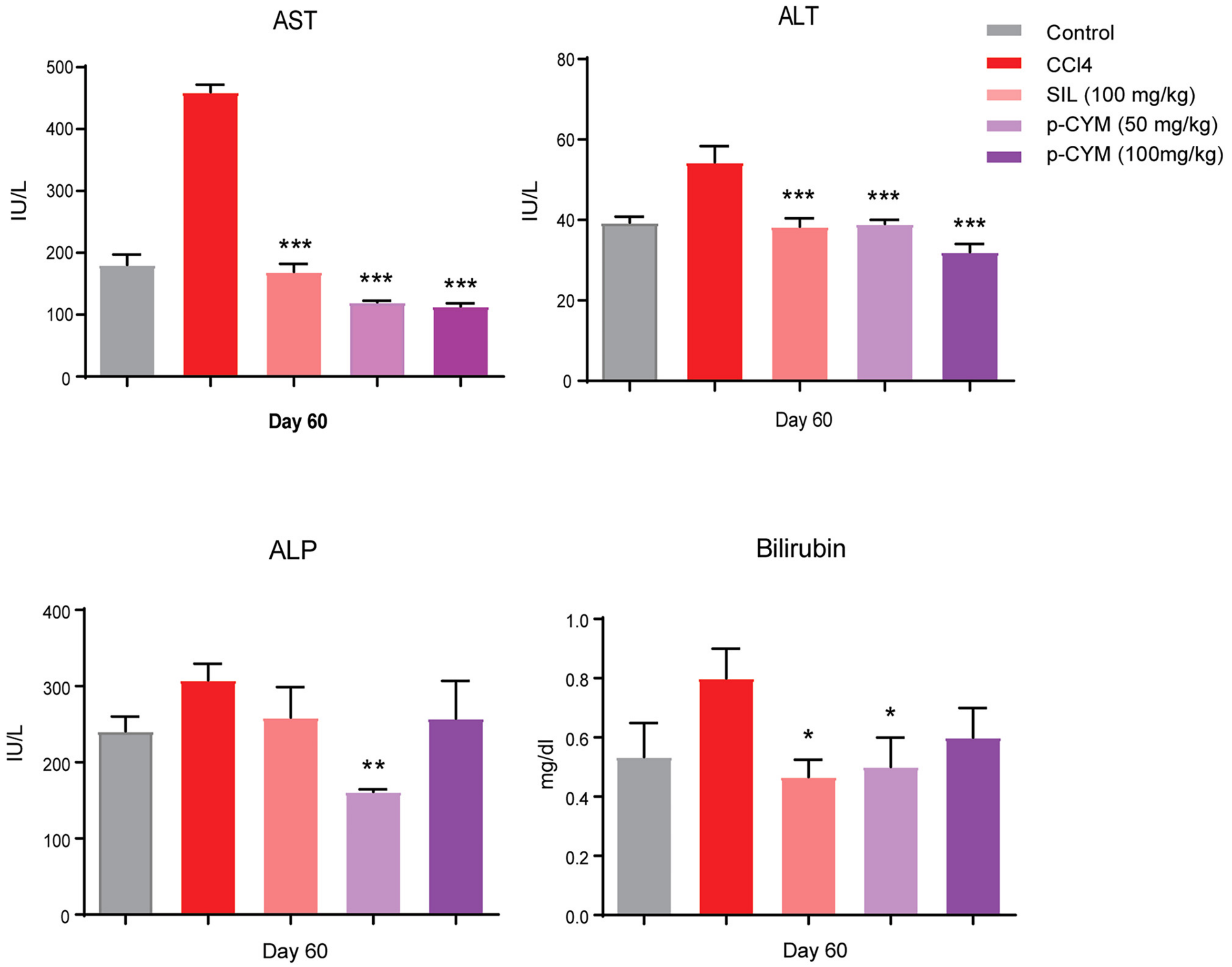

Figure S5: p-CYM reduced CCl4 induced LFTs. LFTs were reduced by the treatment groups while disease group showed a marked induction as compared to control group. ***≤0.001, **≤0.01, *≤0.05 (One-way ANOVA followed by Tukey's multiple comparison test; *n* = 4).

Table S1: List of primers used for qPCR

Gene	Name	Sequence (5′–3′)	BP	T_m (°C)	Species
IL-1β	IL-1β F:	CACCTCTCAAGCAGAGCACAG	21	61	Rat
	IL-1β R:	GGGTTCCATGGTGAAGTCAAC	21	59	Rat
TIMP1	TIMP1 F:	AGCAAAAGGCCTTCGTAAAGAC	22	59	Rat
	TIMP1 R:	TGGCTGAACAGGGAAACACT	20	59	Rat
MMP1	MMP1 F:	ACAGTTTCCCCGTGTTTCAG	20	58	Rat
	MMP1 R:	CCCACACCTAGGTTTCCTCA	20	58	Rat
COL1A1	COL1A1 F:	CATGTTCAGCTTTGTGGACCT	21	59	Rat
	COL1A1 R:	GCAGCTGACTTCAGGGATGT	20	60	Rat
TGF-β1	TGF-β1 F:	ATACGCCTGAGTGGCTGTC	19	59	Rat
	TGF-β1 R:	GTTTGGGACTGATCCCATTGAT	22	58	Rat
HPRT	HPRT F:	CTCATGGACTGATTATGGACAGGAC	25	61	Rat
	HPRT R:	GCAGGTCAGCAAAGAACTTATAGCC	25	62	Rat
HPRT	HPRT F:	GAACGTCTTGCTCGAGATGTG	21	60	Human
	HPRT R:	CCAGCAGGTCAGCAAAGAATT	21	60	Human
IL-6	IL-6 F:	ACCTGAACCTTCCAAAGATG	20	60	Human
	IL-6 R:	GCTTGTTCCTCACTACTCTC	20	60	Human
TNF-α	TNF-α F:	ACCCTCTCTCCCCTGGAAAGGACA	24	60	Human
	TNF-α R:	TGAGGAACAAGCACCGCCTGGA	22	60	Human
COL1A1	COL1A1 F:	AAGCAACCCAAACTGAACCC	20	60	Human
	COL1A1 R:	TTCAAGCAAGTGGACCAAGC	20	60	Human
MMP1	MMP1 F:	CTGGCCACAACTGCCAAATG	20	60	Human
	MMP1 R:	CTGTCCCTGAACAGCCCAGTACTTA	25	60	Human
TIMP1	TIMP1 F:	CCTTCTGCAATTCCGACCTC	20	60	Human
	TIMP1 R:	GTATCCGCAGACACTCTCCA	20	60	Human
TGF-β1	TGF-β1 F:	TTGAGACTTTTCCGTTGCCG	20	60	Human
	TGF-β1 R:	CGAGGTCTGGGGAAAAGTCT	20	60	Human
GPX7	GPX7 F:	AACTGGTGTCGCTGGAG AAG	20	60	Human
	GPX7 R:	AAACTGGTTGCAGGGGA AG	19	60	Human

Table S2: Semi-quantitative scoring of histopathological sections

Groups	Inflammation	Fibrosis	Hepatocytes swelling
Control	–	–	–
CCl4	+	+	+
p-CYM (50 mg/kg)	–	–	+
p-CYM (100 mg/kg)	–	–	–
SIL (100 mg/kg)	–	–	–

−: Absent; +: Mild, ++: Moderate, +++: Severe.
